# High-performance simplification of triangular surfaces using a GPU

**DOI:** 10.1371/journal.pone.0255832

**Published:** 2021-08-05

**Authors:** Mohamed H. Mousa, Mohamed K. Hussein

**Affiliations:** 1 Department of Computer Science, Faculty of Computers and Informatics, Suez Canal University, Ismailia, Egypt; 2 Department of Information Technology, College of Computer Science at AlKamil, University of Jeddah, Jeddah, Saudi Arabia; University of Management and Technology, PAKISTAN

## Abstract

Due to advances in high-performance computing technologies, computer graphics techniques—especially those related to mesh simplification—have been noticeably improved. These techniques, which have a strong impact on many applications, such as geometric modeling and visualization, have been well studied for more than two decades. Recent advances in GPUs have led to significant improvements in terms of speed and interactivity. In this paper, we present a mesh simplification algorithm that benefits from the parallel framework provided by recent GPUs. We customize the halfedge data structure for adaption with the dynamic memory restrictions of CUDA. The proposed algorithm is fully parallelized by employing a lock-free skip priority queue and a set of disjoint regions of the mesh. The proposed technique accelerates the simplification process while preserving the topological properties of the mesh. Some results and comparisons are provided to verify the efficiency of the proposed algorithm.

## Introduction

With the recent improvements in high-performance computing (HPC) technologies, notable progress has been made in many fields, particularly in the computer graphics domain. Tasks in the computer graphics domain depend on piecewise linear surfaces, and with the increasingly strict requirements for quality, models with surfaces that contain hundreds of thousands or millions of polygons can be encountered. Triangles are commonly employed polygons for these surfaces, and the triangular mesh is one of the preferred boundary representations. In general, these meshes have a finite number of triangles and a certain surface resolution. Therefore, to overcome discretization issues, these meshes frequently contain a large number of triangles. Modern scanners and computer-aided designs can be utilized to obtain complex meshes. However, isosurface extraction techniques generate uniform and high-density meshes. These meshes are employed in many applications, such as surface rendering and visualization [1] and virtual reality [2].

Because of the restrictions of graphical devices, meshes are not easy to render and manipulate [3, 4]. Moreover, there is a tradeoff between the accuracy of a mesh and the processing time. Therefore, in addition to improving the capabilities of hardware, efficient decimation algorithms are urgently needed [5, 6]. The principal objective of decimation techniques is to minimize the number of triangles in the graphical pipeline such that the visual difference is minimized; these techniques comprise a preprocessing phase. Mesh simplification has been intensively explored for many decades [7, 8]; however, recent improvements in the computational power of HPC graphics processing units (GPUs) has shifted research to new and promising areas [9–13].

### Contribution

**Fig 1:**
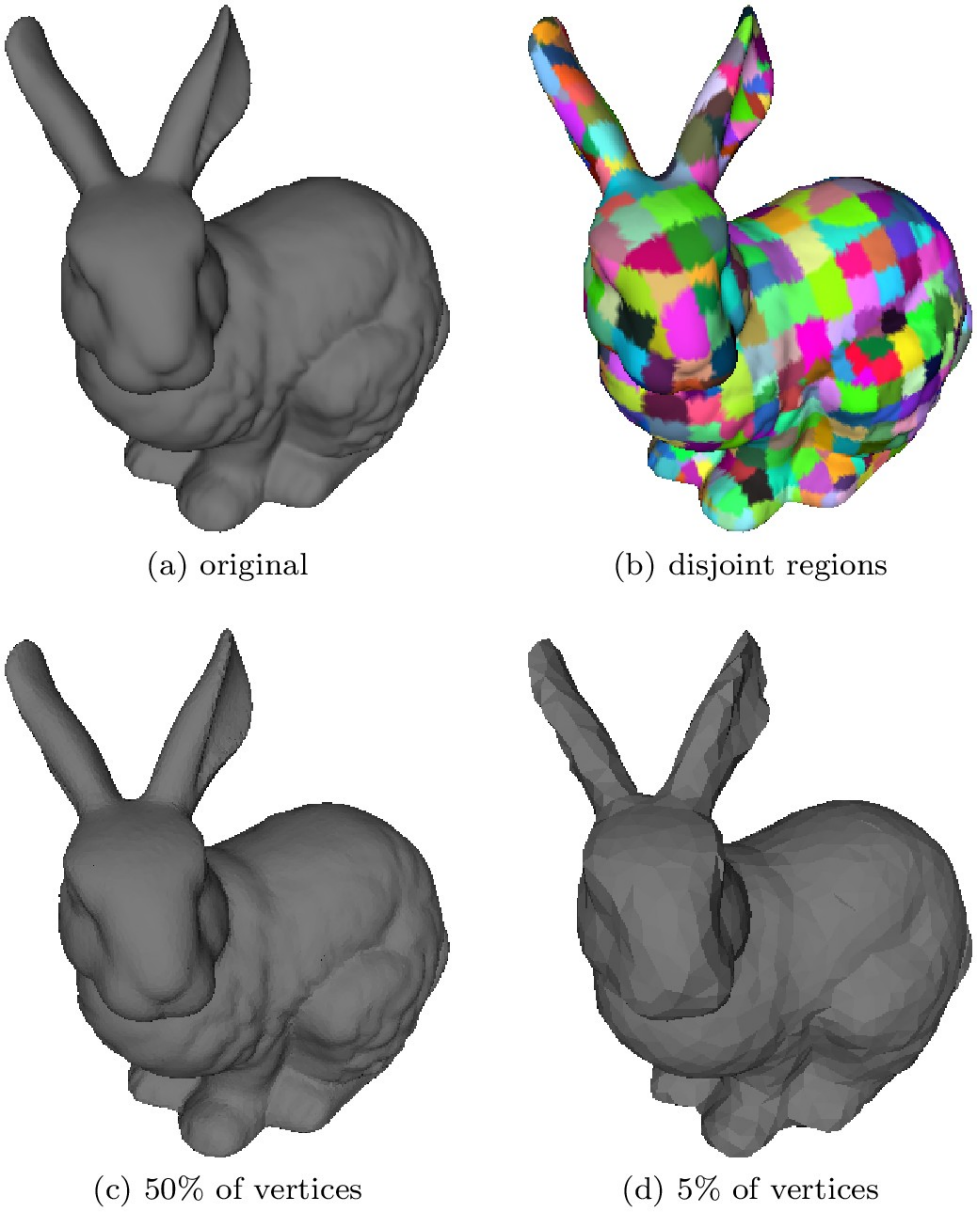
Overview of the proposed simplification process. (a) Stanford bunny, and (b) identification of disjoint parts; (c), (d) simplified versions of the mesh. The percentage here reflects the number of vertices with respect to the number of vertices of the original model.

In this paper, we present a parallel algorithm for mesh decimation. The proposed algorithm relies on recent GPU advances and the efficient parallel computing platform offered by CUDA [14]. We have accelerated surface simplification by parallelizing elementary operations (i.e., edge contraction). Our proposed algorithm is summarized in (Fig 1), which shows the four basic steps of the algorithm.

1. A customized halfedge structure is developed to maintain the geometric and topological information in the GPU. Storing the data structure entirely in the device eliminates the memory overhead required to access the mesh during simplification.
2. A lock-free skip list is used as a customized priority queue to manipulate the list of candidate halfedges. These halfedges are ordered according to their contraction cost [15]. The use of this kind of synchronized queue avoids problems related to the race condition of the concurrent threads manipulating the mesh.
3. A set of independent regions of the given mesh is constructed to enable the edge contraction operations along these regions to run in parallel. In fact, our partition scheme creates a balanced set of partitions even if the input meshes vary in terms of density. Therefore, by updating the topological information of the local neighborhoods after a set of parallel halfedge contraction operations on independent areas, the topological consistency of the simplified mesh is maintained.
4. A set of edge contraction operations is performed in parallel until the global stopping condition (usually a target number of vertices) is reached.

The proposed algorithm has the following advantages:

- It can remove many edges in parallel at a reduced time cost compared to other serial and parallel algorithms.
- It selects global minimum candidates for simplification, thereby guaranteeing the best geometric quality.

The remainder of this article is structured as follows: “Related work” presents a brief review of related research. “The proposed algorithm” starts with an overview of the proposed simplification algorithm, followed by a description of the data structure and quadric error metric (QEM) calculation in “Data structure” and “Halfedge contraction cost”, respectively. The extended search list is discussed in “Customized priority queue”, while the spatial partitioning of the mesh is given in “Disjoint partitioning”. We present our results in “Result and comparisons” and conclude the paper in “Conclusion”.

## Related work

Various mesh simplification techniques have been proposed [5, 7, 16–20], and the HPC architectures of recent GPUs have inspired parallel techniques [9, 12, 13] that can be applied to effectively simplify meshes. In the following two subsections, we summarize the key research on serial and parallel methods.

### Serial approaches

Serial approaches use iterative methods to simplify input meshes. These iterative methods are classified into two main groups: vertex clustering and edge contraction. The key differences between the two groups are as follows:

1. the selection of the candidate element to delete from the mesh;
2. the repositioning of the new or remaining elements after deletion.

The vertex clustering technique, which was first introduced by [21], voxelizes the mesh into *n*^3^ voxels, where *n* is the dimension of the grid. Each nonempty voxel is replaced with a candidate vertex that represents the contained vertices [22]. Vertex clustering techniques are very fast and have high decimation rates. However, these techniques produce low-quality simplified meshes [7], as voxelization does not preserve the salient features of the surface.

In contrast, edge contraction techniques are based on the iterative application of a combinatorial operation that is referred to as edge contraction. This operation merges the two endpoints of a given edge into a new vertex by deleting one vertex and two (or one) triangles at a time. [7] introduced a simplification algorithm named QSlim that associates a geometric error with each edge contraction operation. Low-cost edge contraction enables the production of high-quality simplified meshes [23]. Simplification techniques using the QEM are a compromise between two types of methods that are very fast but have poor quality and are very slow but have high quality. Thus, both speed and quality are considered [24]. [25] described the vertex clustering operation as a set of successive edge contraction operations of certain vertices. A similar approach was proposed by [26]; their decimation algorithm is based on triangle collapse operations. However, these operations require many instances to be manipulated during an application.

### Parallel approaches

Due to recent GPU advances, several techniques have aimed to transform serial approaches into parallel frameworks [10, 12, 13, 27]. However, early approaches focused on concurrency rather than quality [9].

Concerning the vertex clustering techniques, [9] implemented classic vertex clustering on GPUs using geometry shaders. They developed a fast and parallel octree vertex clustering method. However, the proposed approach suffers from the same quality problems faced by all clustering techniques. The quality of the mesh is based mainly on the cell dimension utilized during voxelization. [10, 28] distributed the simplification process between a central processing unit (CPU) and a GPU. Swapping the execution between a CPU and a GPU causes high memory overhead since data must be frequently transferred between these components.

Regarding edge contraction techniques, [29] adopted a progressive mesh data structure [17] to be executed on a GPU, and [30] proposed an iterative parallel GPU-based edge contraction simplification technique. However, the identification of independent parts is not straightforward. In addition, postprocessing sorting and topological updates are applied after each group of steps, which increases the processing time. Moreover, [13] implemented a full-side iterative GPU simplification for triangular meshes that was faster than classic CPU iterative techniques. Similarly, [12] introduced an iterative algorithm that was faster than the method of [13]. However, the iterative nature of the two algorithms slows the simplification process. In addition, the update of the QEM at each vertex after removal is not applicable. [31] proposed a probabilistic selection scheme for edge contraction. They reduced the amount of required memory by choosing to not use a global cost list for the ordered edges. However, the selection of edges with the global minimum is not guaranteed, which affects the overall mesh quality.

## The proposed algorithm

Our proposed algorithm mainly benefits from recent advances in CUDA [14]. The algorithm can be summarized as a set of parallel simplification CUDA kernels that work on independent areas of the mesh. The simplification process is summarized in the following four steps:

1. Adapt a halfedge data structure to maintain the input mesh entirely in the GPU memory.
2. Calculate the edge contraction cost, the QEM, for each halfedge.
3. Build a customized priority queue via a skip list of these costs to choose the candidate pair of vertices to be contracted.
4. Construct the set of disjoint parts of the mesh using k-d tree space partitioning.
5. Apply the decimation process.

In the following subsections, we describe each step and their implementation issues.

### Data structure

This subsection describes the proposed customization of the halfedge data structure. CUDA does not currently support dynamic memory management for memory spaces that are allocated in the global memory shared between the host and device sides. However, vertices, halfedges and triangles are dynamically created and deleted during simplification. To overcome this issue, we have customized the halfedge data structure [32] for adaption to CUDA memory management restrictions.

**Fig 2:**
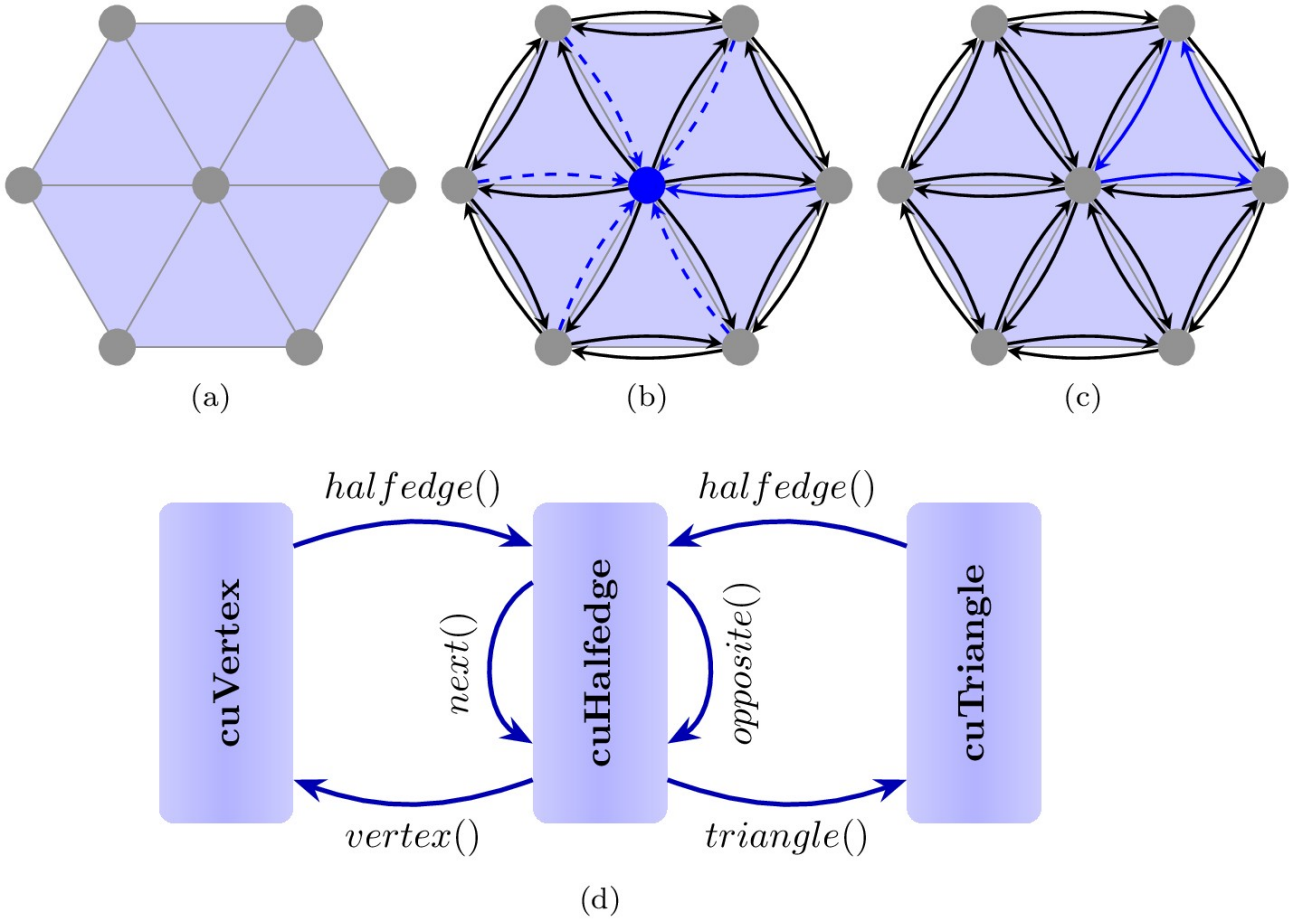
The halfedge data structure. (a) A triangular mesh, (b) the vertex is identified by one of its incident halfedges, (c) the triangle is identified by its three incident halfedges, and (d) the design of the data structure reflecting the relationship between the three components.

Our customized halfedge data structure is based on three main substructures: cuVertex, cuHalfedge and cuTriangle, as shown in (Fig 2). The input mesh is passed to the pipeline as two vectors; from the vertices and triangles, we build an array of halfedges. Each cuVertex provides the following information:

- the geometric coordinates of the vertex (12 bytes);
- a handle for one of its incident halfedges (4 bytes);
- a handle for the container partition (see the “Disjoint partitioning” section) (4 bytes);
- its QEM (see the “Halfedge contraction cost” section) (44 bytes).

Similarly, each cuHalfedge provides the following information:

- a handle for its incident cuVertex (4 bytes);
- a handle for its incident cuTriangle (4 bytes);
- a handle for its opposite cuHalfedge (if it exists) (4 bytes);
- a handle for the next cuHalfedge in the incident triangle (4 bytes);
- its contraction cost (4 bytes).

**Fig 3:**
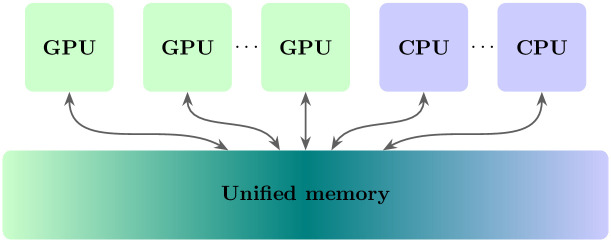
The unified memory.

Each cuTriangle provides a handle for one of its three incident halfedges (4 bytes). The whole structure should reside in the GPU memory and be allocated from the CPU host side. Moreover, if we have more than one GPU, then the data structure should be accessible for all the GPUs’ kernels in addition to creating the CPU. The only solution is the use of CUDA’s page migration engine, i.e., “cudaMallocManaged()”, which supports the unified memory principle, as shown in (Fig 3). The unified memory provides a single address space that is reachable from any CPU or GPU in the system. The unified memory may cause memory overhead when migrating page tables from one physical memory to another physical memory. To overcome this issue, we transfer all required data to the GPU memory before launching the processing kernels using the CUDA application programming interface (API), “cudaMemPrefetchAsync()”. In our experiments, we use a single GPU; however, the application on multiple GPUs is straightforward.

Now, we will describe how to build the vertex, halfedge and triangle relationships. (Algorithm 1) describes how to build the cuHalfedge data structure from lists of vertices and triangles. The first loop fills in the triangle and vertex for every halfedge. The first loop also assigns the halfedge attribute for the associated triangles and vertices. The second and third loops build the opposite relationships between related halfedges.

**Fig 4:**
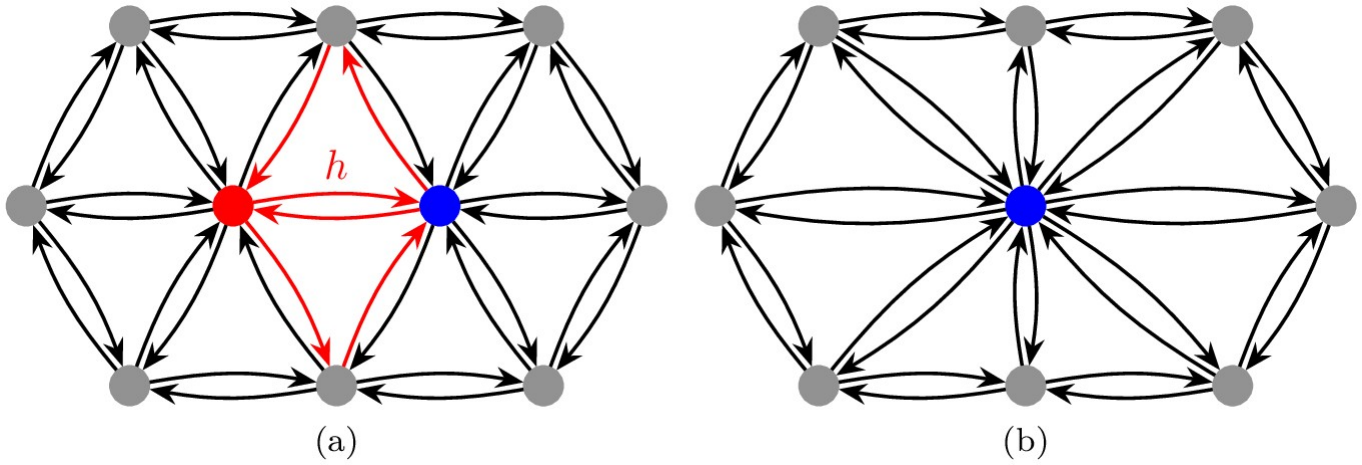
The edge contraction operation. (a) The two candidate vertices, and (b) the mesh after contracting the corresponding edge. The colored halfedges (red) and their incident triangles are marked as deleted.

For the contraction operation, a pair of vertices is tested before contraction to ensure that it does not violate the manifold properties of the mesh. Edge contraction is applied as depicted in (Fig 4). The vertex *h*.*opposite*().*vertex*() is deleted, and *h*.*vertex*() becomes the new vertex. Once the halfedge is contracted, the incident triangles and their halfedges are deleted, and the opposite relationships between the other halfedges around the new vertex are updated. On the other hand, since the data are allocated in the GPU global memory from the CPU host side, the memory occupied by the mentioned arrays cannot be dynamically freed or resized during simplification. To overcome this issue, we associate a flag with each vertex, halfedge and triangle to indicate whether the entry has been previously deleted from the data structure. To accelerate memory access, the deleted items are moved to the end of their container arrays as described in (Algorithm 2). The contraction steps are summarized in (Algorithm 3).

**Algorithm 1: Build the data structure**.

**input**: *T*, *V*

**Output**: H

**for** *i* = 0 ⋯ #*T* **do** in parellel

 *H*[3*i* + *k*] ⋅ *triangle* = *i*;                // (*k* = 0 ⋯ 2)

 *H*[3*i* + *k*] ⋅ *next* = *H*[3 *i* + (*k* + 1)%3];         //(*k* = 0 ⋯ 2)

 *H*[3*i* + *k*] ⋅ *vertex* = *T*[*i*][*k*];              //(*k* = 0 ⋯ 2)

 *T*[*i*][*k*] ⋅ *halfedge* = *H*[3*i* + *k*];            //(*k* = 0 ⋯ 2)

 *T*[*i*] ⋅ *halfedge* = *H*[3*i*];

**end**

// Build the opposite relationships

**for** *i* = 0 ⋯ #*H* **do**

 // Build a list of incident halfedges for each vertex

 *H*_*tmp*_[*H*[*i*] ⋅ *vertex*] ⋅ *append*(*H*[*i*]);

**end**

**for** *i* = 0 ⋯ #*V* **do** in parallel

 **for** *j* = 0 ⋯ *H*_*tmp*_[*i*].*size* **do**

  **for** *k* = 0 ⋯ *H*_*tmp*_[*i*].*size* and *k* ≠ *j* **do**

   *h*_1_ = *H*_*tmp*_[*i*][*j*];

   *h*_2_ = *H*_*tmp*_[*i*][*k*];

   **if** *h*_2_ ⋅ *next* ⋅ *vertex* ∈ *h*_1_ ⋅ *triangle* **then**

    *h*_1_ ⋅ *opposite* = *h*_2_ ⋅ *next*;

    *h*_2_ ⋅ *next* ⋅ *opposite* = *h*_1_;

   **end**

  **end**

 **end**

**end**

**Algorithm 2: The functions deleteVertex, deleteHalfedge and deleteTriangle replace the corresponding item by the last vertex, halfedge and triangle in the list, respectively**.

**Function** *deleteVertex(cuVertex v)*

 *tmp* ← last vertex;

 *v* ← *tmp*;

 *h* ← *v* ⋅ *halfedge*;

 **repeat**

  *h* ⋅ *vertex* ← *v*;

  *h* ← *h* ⋅ *next* ⋅ *opposite*;

 **until** *h* ≠ *v* ⋅ *halfedge*;

 mark *tmp* as deleted;

**end**

**Function** *deleteHalfedge(cuHalfedge h)*

 *tmp* ← last halfedge;

 *h* ← *tmp*;

 *h* ⋅ *next* ⋅ *next* ⋅ *next* ← *h*;

 *h* ⋅ *opposite* ⋅ *opposite* ← *h*;

 mark *tmp* as deleted;

**end**

**Function** *deleteTriangle(cuTriangle t)*

 *tmp* ← last triangle;

 *t* ← *tmp*;

 *t* ⋅ *halfedge* ⋅ *triangle* ← *t*;

 *t* ⋅ *halfedge* ⋅ *next* ⋅ *triangle* ← *t*;

 *t* ⋅ *halfedge* ⋅ *next* ⋅ *next* ⋅ *triangle* ← *t*;

 mark *tmp* as deleted;

**end**

**Algorithm 3: The steps of the halfedge contraction**.

**Function** *contractHalfedge(cuHalfedge h)*

 deleteVertex(*h* ⋅ *opposite* ⋅ *vertex*);

 deleteTriangle(*h* ⋅ *face*);

 deleteTriangle(*h* ⋅ *opposite* ⋅ *face*);

 /* *update the incident property of halfedges around the remaining vertex*: *h*⋅*vertex* */

 *tmp* ← *h* ⋅ *opposite*;

 **repeat**

  *tmp* ⋅ *vertex* ← *h* ⋅ *vertex*;

  *tmp* ← *tmp* ⋅ *next* ⋅ *opposite*;

 **until** *tmp* ← *h* ⋅ *opposite*;

 /* *update the opposite property between the remaining halfedges* */

 *h*1 ← *h* ⋅ *next* ⋅ *opposite*;

 *h*2 ← *h* ⋅ *next* ⋅ *next* ⋅ *opposite*;

 *h*3 ← *h* ⋅ *opposite* ⋅ *next* ⋅ *opposite*;

 *h*4 ← *h* ⋅ *opposite* ⋅ *next* ⋅ *next* ⋅ *opposite*;

 *h*1 ⋅ *opposite* ← *h*2;

 *h*2 ⋅ *opposite* ← *h*1;

 *h*3 ⋅ *opposite* ← *h*4;

 *h*4 ⋅ *opposite* ← *h*3;

 deleteHalfedge(*h*);

 deleteHalfedge(*h* ⋅ *next*);

 deleteHalfedge(*h* ⋅ *next* ⋅ *next*);

 deleteHalfedge(*h* ⋅ *opposite*);

 deleteHalfedge(*h* ⋅ *opposite* ⋅ *next*);

 deleteHalfedge(*h* ⋅ *opposite* ⋅ *next* ⋅ *next*);

**end**

### Halfedge contraction cost

In this section, we describe how to evaluate the QEM associated with each vertex. A plane is identified by the equation ***n*** ⋅ ***p*** + *d* = 0, where:

- *n* = (*n*_*x*_, *n*_*y*_, *n*_*z*_) is the unit normal, i.e., a direction perpendicular to the plane such that $n_{x}^{2}+n_{y}^{2}+n_{z}^{2}=1$, and
- *d* is an offset scalar representing the signed distance from the origin to the plane.

For any point that does not lie on the plane, the expression ***n*** ⋅ ***p*** + *d* represents the signed distance from the given point *p* to the plane. The squared distance can be evaluated by

$$\begin{array}{c} D\left(p\right)={\left(n_{x}x+n_{y}y+n_{z}z+d\right)}^{2} \end{array}$$ (1)

This equation can be rewritten in matrix form as follows:

$$\begin{array}{c} D\left(p\right)=(p^{t}(nn^{t})p+2{\left(dn\right)}^{t}p+d^{2}) \end{array}$$ (2)

The quadric of the plane, ***n*** ⋅ ***p*** + *d* = 0, is defined as *Q* = (*q*_1_, *q*_2_, *q*_3_), where:

- *q*_1_ = ***nn***^*t*^ is a 3 × 3 symmetric matrix,
- *q*_2_ = *d**n*** is a vector, and
- *q*_3_ = *d*^2^ is a scalar.

Therefore, given any quadric, the squared distance is evaluated by

$$\begin{array}{c} Q\left(p\right)=p^{t}q_{1}p+2q_{2}^{t}p+q_{3} \end{array}$$ (3)

Each vertex of the mesh is associated with a set of planes that correspond to the incident triangles. The sum of the squared distances is given by the following equation:

$$\begin{array}{c} Q\left(v\right)=\sum_{i}Q_{i}\left(v\right) \end{array}$$ (4)

The linearity of the *Q* operator makes the sum of a set of quadrics equal to the sum of the corresponding components of the triples $\left(q_{1}^{i},q_{2}^{i},q_{3}^{i}\right)$, i.e.,

$$\begin{array}{c} Q\left(v\right)=\sum_{i}Q_{i}\left(v\right)=(\sum_{i}q_{1}^{i},\sum_{i}q_{2}^{i},\sum_{i}q_{3}^{i})\left(v\right) \end{array}$$ (5)

The triangle plane may be shared with at least three vertices. Therefore, to remove computational redundancy, a set of CUDA kernels is executed to calculate the plane equation and then the quadrics of the triangles of the mesh. Similarly, a set of CUDA kernels is executed to sum the corresponding quadric for each vertex. Hence, the contraction cost of a halfedge *h* is determined by the following steps:

1. Determine the vertex *v*_*i*_ = *h*.*vertex*().
2. Determine the vertex *v*_*j*_ = *h*.*opposite*().*vertex*().
3. Determine the position of the new vertex *v*.
4. Evaluate the new quadric *Q* = *Q*_*i*_ + *Q*_*j*_, which is the quadric of the new vertex *v*.
5. Obtain the edge contraction cost *Q*(*v*).

The position of the new vertex *v* should be chosen such that *Q*(*v*) is minimized. This minimization is expressed as ∇*Q*(*v*) = 2*q*_1_ *v* + 2*q*_2_ = 0, which yields $v^{\prime }=-q_{1}^{-1}q_{2}$; its minimization error is expressed as follows:

$$\begin{array}{c} \begin{array}{ccc} Q(v^{\prime }) & = & v^{\prime t}q_{1}v^{\prime }+2q_{2}^{t}v^{\prime }+q3\\ & = & {\left(-q_{1}^{-1}q_{2}\right)}^{t}q_{1}\left(-q_{1}^{-1}q_{2}\right)+2q_{2}^{t}\left(-q_{1}^{-1}q_{2}\right)+q_{3}\\ & = & -q_{2}^{t}q_{1}^{-1}q_{2}+q_{3} \end{array} \end{array}$$ (6)

which is a special case of the standard positive definite quadratic minimization that can be solved by using the Cholesky factorization [33, 34]. During simplification, the halfedges with minimum costs are selected for contraction.

### Customized priority queue

In this section, we describe how to maintain the costs of the set of halfedges in a customized priority queue. Concurrent access to the priority queue during simplification hinders the use of classic data structures for priority queues.

Classically, the implementation of queues using arrays rather than linked lists is recommended. However, lists are better than arrays for the insertion and removal operations. The main drawback of using a sorted linked list is the sequential access of its elements, *O*(*n*), with *O*(lg *n*) for sorted arrays. To overcome this problem, we employ lock-free skip lists to implement our priority queue [35]. Additionally, this skip-list priority queue prevents the synchronization drawbacks that can occur due to race conditions. Moreover, the speed of the search, insertion and removal operations is increased to *O*(lg *n*) due to the multilayer structure.

**Fig 5:**
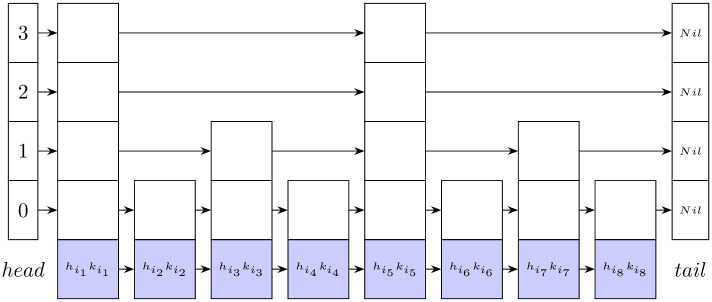
A skip list consisting of 4 linked lists. The original list of pairs (vertices, keys) is at level 0 and sorted in ascending order. Each level contains a sublist from the preceding level.

A skip list is a nondeterministic framework based on linked lists, which consists of a multilevel linked list. The 0-level contains all elements of the list. The subsequent levels contain fewer elements. Each level is a subset of the preceding level and a superset of the next level. In (Fig 5), the original list is the set of halfedges, $h_{i_{j}}$, sorted by their quadric costs $k_{i_{j}}$. The insertion of any halfedge into the skip list starts at level 0, while element removal starts from the top level.

We will now describe how the concurrent insertion and removal of halfedges can be performed safely by using the CUDA atomic function *atomicCAS()* [14]. The *atomicCAS()* function has the following form: int *atomicCAS*(int *address, int *compare*, int *val*). The function loads the value *old* located at *address*, evaluates the expression *(old == compare? val: old)* and saves the results at the location *address*. Thus, *atomicCAS()* ensures that the value at *address* is not changed using other threads. If the value is changed, then the function can easily detect this change.

The two main primitives that we employ are the insert operation and delete operation. To insert a pair (*h*_*i*_, *k*_*i*_)—the halfedge and its quadric cost—into the list, we navigate the list until we obtain the smallest entry (*h*_*j*_, *k*_*j*_) such that *k*_*j*_ > *k*_*i*_. A new entry is created with a value of (*h*_*i*_, *k*_*i*_), and *address*((*h*_*i*_, *k*_*i*_)).*next* is set to *address*((*h*_*j*_, *k*_*j*_)). Next, *atomicCAS()* is applied to set *address*((*h*_*j*_, *k*_*j*_)).*previous*.*next* to *address*((*h*_*i*_, *k*_*i*_)). Furthermore, to remove the pair (*h*_*i*_, *k*_*i*_) from the list, *atomicCAS()* is applied to set *address*((*h*_*i*_, *k*_*i*_)).*previous*.*next* to *address*((*h*_*i*_, *k*_*i*_)).*next*. The node that contains (*h*_*i*_, *k*_*i*_) is not deleted due to CUDA restrictions but is instead added to a deleted list for further usage. During simplification, the halfedge nodes, for which the costs must be updated, are simply deleted, and the new values are inserted. (Algorithm 4) shows how the Find function determines the appropriate position of the halfedge with respect to its cost in the skip list for further insertion or deletion.

**Algorithm 4: The Find function determines the appropriate position of** *key* **in the skip list**.

**Function** *Find(key)*

 *tmp* ← top-first element in the skip list;

 // step down

 **while** *tmp*.*below* ≠ *null* **do**

  *tmp* ← *tmp*.*below*;

  // step forward

  **while** *key* ≥ *tmp*.*next*.*cost* **do**

   *tmp* ← *tmp*.*next*;

  **end**

 **end**

 **return** *tmp*;

**end**

### Disjoint partitioning

Once the priority queue is constructed for the given triangular mesh, we must construct a set of independent areas on which we will parallelize the simplification process. In this section, we describe the major steps for constructing a set of disjoint partitions of the mesh using the k-d tree.

**Algorithm 5: Space partitioning of** M **using the k-d tree**.

root←AABB(M) ;

*SplitList* ← *root*;

**repeat**                 // parallel

 *tmp* ← *Pop*(*SplitList*);

 **if** *C*(*tmp*) **then**

  (*leftchild*, *rightchild*) ← *Split*(*tmp*);

  *SplitList*.*append*(*leftchild*);

  *SplitList*.*append*(*rightchild*);

 **end**

**until** *SplitList is empty*;

The partitioning procedure is summarized in (Algorithm 5). The algorithm starts by determining the axis-aligned bounding box of the mesh M. The initial bounding box is the root of the k-d tree. Starting from this root, the following steps are applied for the candidate node:

1. Evaluate the splitting condition, *C*(*P*_*i*_), which in our case is a fixed number of contained points, i.e.,
  $$\begin{array}{c} C\left(P_{i}\right)=\{\begin{array}{cc} true & |P_{i}|\leq \zeta \\ false & otherwise \end{array} \end{array}$$ (7)
  where *ζ* is the desired number of points in each cell *P*_*i*_. This splitting condition generates a balanced number of partitions, even if the input mesh is not uniformly sampled.
2. If *C*(*P*_*i*_) is satisfied, then *P*_*i*_ is appended to the split queue, *SplitList*.
3. If *C*(*P*_*i*_) is not satisfied, then *P*_*i*_ is a leaf node and is removed from the *SplitList* queue.

### Simplification process

In the previous sections, we showed how to build the priority queue and identify the independent regions. Now, the simplification process is ready for execution in parallel. Since the contraction operator changes the topological properties of the mesh, we allow a single halfedge contraction for each independent region of the mesh at a time, which guarantees the consistency of the neighborhood properties among the vertices of the mesh.

Without loss of generality, our stopping criterion is that the mesh must be simplified to a certain number of vertices. Note that each contraction process reduces the total number of vertices by 1. The simplification process starts by creating the set of threads *T*. *T* picks the top halfedges from the customized priority queue, i.e., the halfedges with the minimum cost. For each independent region *P*_*i*_, we create the thread *T*_*i*_. *T*_*i*_ picks a halfedge *h* from the top of the priority queue. If *h* ∉ *P*_*i*_, then the halfedge is skipped, and *T*_*i*_ picks the next candidate halfedge. However, if only one of the two endpoints belongs to *P*_*i*_, then the halfedge is selected, and the neighbor partition is blocked. Thus, the halfedge that crosses two adjacent partitions is contracted by the first partition thread, and the other partition thread waits until this halfedge is contracted, which avoids any overlap of the topological update of the conflicting areas.

However, a thread does not execute the contraction operation unless the halfedges of the smaller costs are picked from the queue for contraction or the total number of picked and skipped halfedges does not reach the target number of vertices. This condition gives a higher priority to contraction of halfedges with less cost to minimize the global geometric error. The simplification process is summarized in (Algorithm 6).

**Algorithm 6: Major steps of the proposed algorithm**.

Calculate the QEM for the set of halfedges // parallel

Build the customized priority queue // parallel

Construct the independent partitions {*P*_*i*_} // parallel

*skipped* ← 0;                // shared

*removed* ← 0;              //shared

**for each** *P*_*i*_ **do**              // parallel

 pick a halfedge *h*;

 **while** *h* ∉ *P*_*i*_ **do**

  **if** *h* ∩ *P*_*i*_ ≠ *ϕ* **then**

   find *P*_*j*_ such that *h* ∩ *P*_*j*_ ≠ *ϕ*;

   **wait**(*P*_*j*_, *h* is contracted);

  **end**

  *skipped* ← *skipped* + 1;

  pick another *h*;

 **end**

 **wait**(*P*_*i*_, *removed* + *skipped* < *target*);

 contract *h*;

 **if** *removed* = *target* **then**

  exit;

 **end**

**end**

As mentioned, the proposed simplification process guarantees decimation of the edges with the lowest costs. Therefore, the set of edges to be contracted will be identical to that of QSlim. The only difference is that these edges will be contracted in a different order, which will not affect the quality measure since the error metric is a linear operator.

## Results and comparisons

We implement the algorithms presented in this paper by using NVIDIA’s CUDA framework [14] on an Intel Core i7–8565U CPU @(1.80 GHz,1.99 GHz) with a GeForce MX130 4GB GPU on Windows 10. Recent architectures of CUDA, especially 5.0, enable direct memory access between the host and the kernel codes. In all algorithms described in this paper, statements that are said to be run in “parallel” are executed as parallel GPU device kernels. Otherwise, they are implemented in serial mode. Considering some CUDA driver limitations, we do not allow device threads to run for more than 8 s. CUDA assumes that device kernels have a short execution time. In cases in which a device kernel reaches its execution time limit, we stop the kernel and regenerate another set to complete the job.

**Fig 6:**
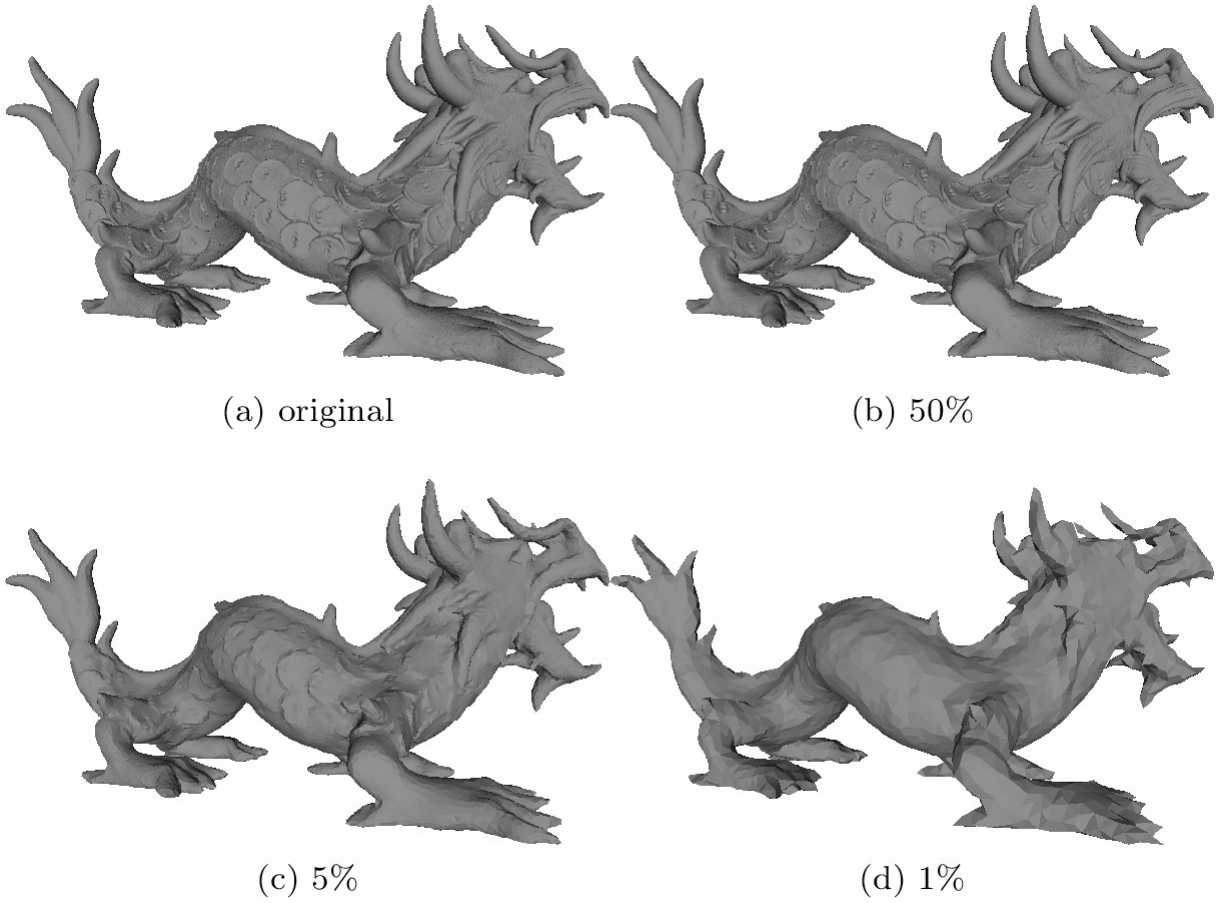
The simplification of the Dragon into different levels of detail. (a) The original dataset, (b) 50% of vertices, (c) 5% of vertices, and (d) 1% of vertices.

The geometric models underlying the results presented in this paper can be found in [36], see “S1 Text”. (Table 1) presents the numbers of points and triangular faces of the meshes utilized in our experiments. We simplified the input models to a target number of vertices. (Fig 6) shows the simplification of the Dragon into levels of detail of 1%, 5% and 50%. The simplification process creates a smooth surface for each simplified version.

**Table 1:** The set of meshes used in our experiments.

Model	#Points	#Faces
Bunny	37000	73996
Gargoyle	863210	1,726,416
Dragon	3609455	7,218,906
Lucy	14,027,872	28,055,742

**Fig 7:**
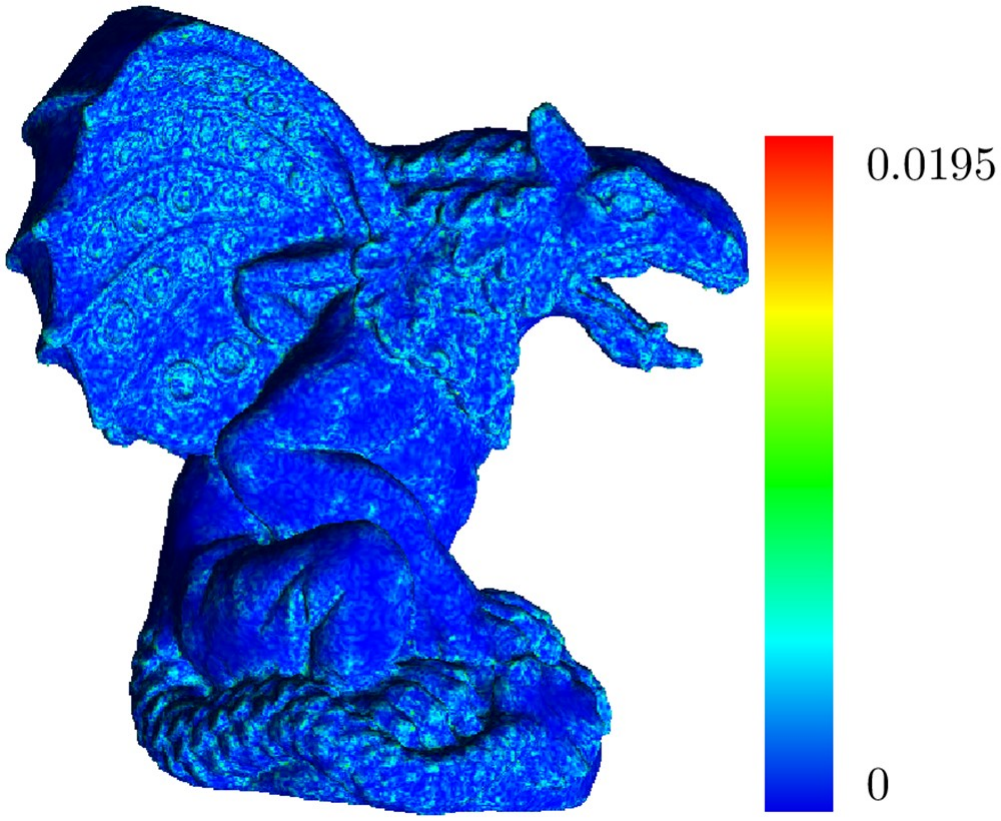
The geometric deviation of a simplified version of the Gargoyle model at 10% of its original number of vertices.

To prove the effectiveness of the proposed approach, we compare our results with those of the QSlim serial algorithm [7] implemented by MeshLab v2016.12 [37] and the MCS GPU algorithm [31]. In addition, we use the geometric deviation, as shown in (Fig 7), as the quality measure [38]. The geometric deviation evaluates the geometric differences between two meshes, in our case, the input and simplified meshes. This is formally defined as follows [39]:

$$\begin{array}{c} \max_{p\in M_{1}}\{d(p,M_{2})\} \end{array}$$ (8)

where *M*_1_ is the input mesh, *M*_2_ is the simplified mesh and *d* is the Euclidean distance. The timing and quality measures of [31] are extracted from the associated paper. (Table 2) shows the times and geometric qualities for the simplification process applied to some of the models of (Table 1) with respect to the mentioned vertex targets. (Table 2) shows that the rate of decimation of vertices per second decreases for large meshes; this phenomenon is attributed to the size of the customized priority queue. The construction and search of small priority queues are faster than those of dense priority queues.

**Table 2:** The time expended in seconds and the quality measures of the simplification of the input meshes in comparison with those of QSlim [7] and MCS [31].

Models	Target	QSlim [7]		MCS [31]		ours	
		time	quality	time	quality	time	quality
Gargoyle	589,860	22.86	0.01966	1.30	0.03542	1.05	0.01967
Dragon	2,466,461	109.02	0.0815	6.39	0.0836	4.29	0.0816
Lucy	9,585,712	469.089	0.0186	28.03	0.032	22.05	0.0187

Additionally, we compare our results with the timing and quality of the GPU-based algorithm [12]. As mentioned in “Related work”, [12] applied an iterative approach. In each iteration, a set of parallel edge contraction operations is performed until a target number of vertices is reached. The higher the number of vertices decimated in each iteration is, the lower the quality of the mesh. To improve the mesh quality, the number of decimated vertices should be decreased in each iteration, which will cause a dramatic increase in time. (Table 3) shows the timing and mesh quality of our algorithm and those of [12] at targets of 25%, 10% and 5% using 18 iterations, 29 iterations and 37 iterations, respectively. The timing and quality evaluations are extracted from the related paper.

**Table 3:** Comparison of the timing in seconds and the quality of the simplification of the Gargoyle model using [12] and our proposed algorithm.

Target	[12]		Ours	
	time	quality	time	quality
25%	3.9	0.01524	0.73	0.00253
10%	5.1	0.03357	0.95	0.01366
5%	5.6	0.05961	1.05	0.01967

## Conclusion

In this paper, we introduce an approach for triangular surface simplification using recent GPU advances. Our approach follows the edge contraction framework, which presents a high-quality surface simplification. We build a customized priority queue based on a skip list for the set of candidate halfedges. This skip list enables simultaneous update of the customized priority queue. The proposed approach identifies a set of independent regions on the surface, on which we apply parallelism. The applied parallelism significantly reduces the time required for the overall process. Compared to competing serial and parallel algorithms, the proposed approach is both simple and efficient.

## Supporting information

S1 Text (PDF)
