# Supplementary material for: High-performance simplification of triangular surfaces using a GPU
Source: PLoS One. 2021 Aug 5;16(8):e0255832. doi: 10.1371/journal.pone.0255832 (PMC8341488; doi:10.1371/journal.pone.0255832)
Supplement: S1 Text — (PDF) [file pone.0255832.s001.pdf]

The geometric models underlying the results presented in this paper are available directly from the following links:

1. Bunny:
   http://graphics.stanford.edu/pub/3Dscanrep/bunny.tar.gz
2. Dragon:
   http://graphics.stanford.edu/data/3Dscanrep/xyzrgb/xyzrgb_dragon.ply.gz
3. Lucy:
   http://graphics.stanford.edu/data/3Dscanrep/lucy.tar.gz
4. Gargoyle:
   http://visionair.ge.imati.cnr.it/ontologies/shapes/view.jsp?id=308-Gargoyle_1.7M

Additional geometric models are available at:

- The Shape repository:
  http://visionair.ge.imati.cnr.it/ontologies/shapes/
- The Stanford 3D Scanning Repository:
  http://graphics.stanford.edu/data/3Dscanrep/
